# Data on the chemical properties of commercial fish sauce products

**DOI:** 10.1016/j.dib.2017.10.022

**Published:** 2017-10-14

**Authors:** Mitsutoshi Nakano, Yoshimasa Sagane, Ryosuke Koizumi, Yozo Nakazawa, Masao Yamazaki, Toshihiro Watanabe, Katsumi Takano, Hiroaki Sato

**Affiliations:** a Department of Food and Cosmetic Science, Faculty of Bioindustry, Tokyo University of Agriculture, 196 Yasaka, Abashiri, Hokkaido 099-2493, Japan; b The Organization for the Promotion of International Relationship, 709, 1-1-7 Motoakasaka Minato-ku, Tokyo 107-0051, Japan; c Department of Applied Biology and Chemistry, Faculty of Applied Bioscience, Tokyo University of Agriculture, 1-1-1 Sakuragaoka, Setagaya-ku, Tokyo 156-8502, Japan

**Keywords:** Fish sauce, Chemical property, Salinity, Protein digestion, Fermented foods

## Abstract

This data article reports on the chemical properties of commercial fish sauce products associated with the fish sauce taste and flavor. All products were analyzed in triplicate. Dried solid content was analyzed by moisture analyzer. Fish sauce salinity was determined by a salt meter. pH was measured using a pH meter. The acidity was determined using a titration assay. Amino nitrogen and total nitrogen were evaluated using a titration assay and Combustion-type nitrogen analyzer, respectively.

The analyzed products originated from Japan, Thailand, Vietnam, China, the Philippines, and Italy. Data on the chemical properties of the products are provided in table format in the current article.

**Specifications Table**

**Table:** 

Subject area	Chemistry
More specific subject area	Food Chemistry
Type of data	Table
How data was acquired	Moisture analyzer (MX-50, A&D, Japan)
	Salt meter (B-721, HORIBA, Japan)
	pH meter (D-52, HORIBA)
	Combustion-type nitrogen analyzer (SUMIGRAPH NC-220F, Sumika Chemical Analysis Service, Japan)
Data format	Raw, analyzed
Experimental factors	Pretreatment for the acidity and nitrogen measurements: dilution in distilled water
Experimental features	Solid content analysis in a moisture analyzer.
	Direct measurements of pH and salinity.
	Total acidity determination by basic titration with phenolphthalein as indicator.
	Total nitrogen content determination by elemental analysis.
	Amino nitrogen content determination by formol titration.
Data source location	Tokyo, Fukuoka and Hokkaido, Japan
Data accessibility	All data are presented in this article

**Value of the data**

• The presented data on the chemical properties of 46 commercially available fish sauce products from Japan, Thailand, Vietnam, China, the Philippines, and Italy may be used as a reference for culinary studies of the fish sauces and related products.
• The data will be useful for nutritional assessment of the fish sauce products based on the chemical properties of these products.
• The presented data will allow the prediction of consumer preferences with regard to fish sauce products in each country.

## 1 Data

Fish sauce is a popular condiment on account of its distinctive flavor and taste. It is obtained by mixing fish material with salt, which is subsequently fermented under natural conditions [1]. In Japan, fish sauce is mainly used as a condiment in “Nabe” cuisine, a Japanese-style stew [1]. Among the Southeastern Asian countries, the widest variety of fermented fish products is found in Thailand [2]. In Vietnam, the fish sauce is used for dipping in a wide variety of dishes [3]. In China, fish sauce is used as a substitute for soy sauce in some dishes [1]. Patis, a Philippine fish sauce, is used in a citrus fruit soup [2]. The Italian fish sauce is based on Garum, which is the earliest reported fish sauce highly appreciated in the Roman era [4]. In general, fish sauces have a predominantly salty and umami taste, and distinctive flavor [5]. Therefore, data on the following were generated: fish sauce salinity, determining the salty taste; acidity, which roughly reflects the organic acids associated with the distinctive flavor and sour taste of the fish sauce; and nitrogen, representing the amino acids associated with the umami taste. Data on the chemical properties of 46 commercial fish sauce products produced in several countries (Japan, Thailand, Vietnam, China, the Philippines, and Italy) are presented. The origin and materials of the analyzed fish sauce products are provided in (Table 1). The data on their dried solid content, salinity, pH, acidity, and nitrogen content are shown in Table 2.

**Table 1:** Fish sauces used in this study.

Product ID	Origin	Raw materials
J1	Japan	Soy sauce, Wheat, Dried bonito shavings, Kelp, and Urchin
J2	Japan	Soy sauce, Protein hydrolysate, Saccharide, Dried bonito extract, and Fish sauce
J3	Japan	Soy sauce, Sugar, Mirin,^a^ Salt, Dried bonito, and Oyster extract
J4	Japan	Salt, Mirin,^a^ Sugar, Soy sauce, Roasted flying fish, and Alcohol
J5	Japan	Glucose fructose liquid sugar, Soy sauce, Salt, Dried bonito extract, Mirin^a^, Sugar, and Roasted flying fish
J6	Japan	Soy sauce, fishes, Fermented seasoning, Salt, Glucose fructose liquid sugar, and Seaweeds
J7	Japan	Soy sauce, Sugar, Glucose fructose liquid sugar, Vegetable protein hydrolysate, Dried bonito extract, Mirin,^a^ Bonito extract, Salt, and Yeast extract
J8	Japan	Salt, Sugar, Mirin,^a^ Soy sauce, Roasted flying fish, Fermented seasoning, Roasted flying fish powder, and Alcohol
J9	Japan	Soy sauce, Saccharide, Fructose liquid sugar, Fermented seasoning, extract, Honey, Salt, Roasted flying fish, and Fish sauce
J10	Japan	Soy sauce, Mirin,^a^ Sugar, Salt, Roasted flying fish, Yeast extract, Dried bonito flakes, Kelp extract, and Fish and shellfish extract
J11	Japan	Soy sauce, Sugar, Mirin,^a^ Salt, Dried shrimps, Brewed vinegar, Yeast, extract, Fish and shellfish extract, Kelp extract, and Dried shiitake mushroom
J12	Japan	Soy sauce, Sugar, Mirin,^a^ Dried bonito extract, Salt, Kelp extract, Oyster extract, Yeast extract, Shiitake mushroom extract, and Alcohol
J13	Japan	Soy sauce, Sugar, Roasted flying fish, Salt, Mirin,^a^ Yeast, extract, Brewed vinegar, Dried shiitake mushroom, Kelp, and Fish and shellfish extract
J14	Japan	Soy sauce, Sugar, Bonito extract, Mirin,^a^ Salt, Kelp extract, Alcohol, and Seasoning
J15	Japan	Japanese sandfishes "Hata-Hata" and Salt
J16	Japan	Soy sauce, Sugar, Rice fermented seasoning, Bonito extract, Dried anchovies extracts, Seasoning, and Sweetener
J17	Japan	Soy sauce, Saccharides, Dried flying fish extract, Salt, Seasoning, Sweetener, and Alcohol
J18	Japan	Japanese sandfishes "Hata-Hata" and Salt
J19	Japan	Acetes and Salt
J20	Japan	Deep-sea smelts "Nigisu", Salt, Soybeans, and Barley rice malt
J21	Japan	Squids, Salt, Rice malt, Sake, and Beer yeast extract
J22	Japan	Squid intestines and Salt
J23	Japan	Squid intestines, Salt, and Shochu^b^
J24	Japan	Cods, Barley rice malt, Salt, Squid intestines, Sugar, and Fructose
J25	Japan	Pagrus major, Salt, Defatted soy bean meal, Wheat, Rice, and Alcohol
J26	Japan	Flying fishes, Soybeans and barley rice malt, and Salt
J27	Japan	Tunas, Salt, and Soybeans and barley rice malt
J28	Japan	Cutlass fishes, Salt, and Rice malt
J29	Japan	Anchovies and Salt
J30	Japan	Soy sauce, Dried bonito extract, Sugar, Salt, Yeast extract, Amino acids, Alcohol, Caramel pigment, Acidifier, Acetic acid, and Thiamine
T1	Thailand	Fish extract and Salt
T2	Thailand	Anchovies, Salt, and Sugar
T3	Thailand	Anchovies extract, Salt, Sugar, and Fructose
T4	Thailand	Sardine extract, Salt, and Sugar
T5	Thailand	Anchovies extract, Salt, and Sugar
T6	Thailand	Seafood, Salt, and Sugar
T7	Thailand	Sardine extract and Salt
T8	Thailand	Seafood extract and Salt
T9	Thailand	Sardine, Salt, and Sugar
T10	Thailand	Sardine extract, Salt, and Sugar
T11	Thailand	Fish sauce, Soy sauce product, Fructose, glucose fructose liquid sugar, Yeast extract, and Amino acids
V1	Vietnam	Fish extract and Salt
V2	Vietnam	Sardine and Salt
C1	China	Anchovies, Salt, and Sugar
P1	Philippine	Mackerel
I1	Italy	Anchovies, and Salt

a Rice wine dominantly used for cooking.

b Japanese spirit distilled from sweet potatoes, rice, etc.

**Table 2:** Dried solid content, salinity, pH, acidity, amino nitrogen, and total nitrogen in 46 commercial fish sauce products from Japan, Thailand, Vietnam, China, the Philippines, and Italy.

Origin and ID for the products	Dried solid content (%)	Salinity (%)	pH	Acidity (mL/100 mL)	Amino nitrogen (% (w/v))	Total nitrogen (%)	Amino/total nitrogen (%)
Japan							
J1	27.13 ± 1.05	16	4.48 ± 0.05	15.64 ± 0.31	0.32 ± 0.13	1.08	29.67
J2	27.69 ± 0.47	20	4.62 ± 0.02	8.23 ± 0.12	0.39 ± 0.08	0.78	49.91
J3	32.78 ± 0.22	19	4.61 ± 0.01	12.12 ± 0.05	0.22 ± 0.03	0.75	29.39
J4	21.93 ± 0.13	15	5.54 ± 0.02	1.71 ± 0.02	0.06 ± 0.01	0.32	18.75
J5	20.74 ± 0.31	11	5.08 ± 0.02	3.16 ± 0.02	0.14 ± 0.08	0.34	40.69
J6	24.06 ± 0.38	11	4.87 ± 0	10.87 ± 0.04	0.27 ± 0.11	0.89	30.51
J7	20.66 ± 0.35	17	4.84 ± 0.01	9.16 ± 0.04	0.21 ± 0.16	0.64	32.89
J8	18.22 ± 1.74	24	5.59 ± 0.03	1.38 ± 0.01	0.05 ± 0.03	0.25	19.86
J9	16.12 ± 0.25	15	4.94 ± 0.01	5.07 ± 0.01	0.12 ± 0.06	0.32	37.17
J10	34.97 ± 1.09	25	4.51 ± 0.04	11.20 ± 0.05	0.25 ± 0.05	0.85	29.58
J11	27.27 ± 0.50	24	4.73 ± 0	4.25 ± 0.01	0.11 ± 0.07	0.35	30.99
J12	32.21 ± 4.25	25	4.72 ± 0.04	15.74 ± 0.01	0.26 ± 0.02	1.16	22.44
J13	25.54 ± 0.27	24	4.82 ± 0.02	5.99 ± 0.01	0.20 ± 0.05	0.52	38.80
J14	26.48 ± 0.91	25	4.69 ± 0.03	12.32 ± 0.04	0.38 ± 0.08	0.99	38.30
J15	32.13 ± 0.12	25	5.44 ± 0.05	6.59 ± 0.05	0.80 ± 0.04	1.28	62.32
J16	24.41 ± 0.56	25	4.81 ± 0.01	10.77 ± 0.03	0.34 ± 0.12	0.83	40.88
J17	35.25 ± 0.14	25	4.97 ± 0.03	9.88 ± 0	0.73 ± 0.10	1.06	69.03
J18	24.39 ± 0.25	25	5.97 ± 0.11	1.98 ± 0	0.38 ± 0	0.38	100.00
J19	33.34 ± 0.11	25	5.33 ± 0.07	7.18 ± 0.08	0.92 ± 0.18	1.72	53.43
J20	29.06 ± 0.16	25	4.87 ± 0.03	13.92 ± 0.07	0.94 ± 0.15	1.78	52.71
J21	24.53 ± 0.24	25	5.73 ± 0.04	5.47 ± 0.03	1.01 ± 0.09	1.5	67.39
J22	32.06 ± 0.19	25	5.36 ± 0.05	7.22 ± 0.04	1.3 ± 0.27	1.98	65.63
J23	26.18 ± 0.32	25	5.81 ± 0.01	9.20 ± 0.01	1.16 ± 0.15	1.8	64.61
J24	31.29 ± 0.22	25	5.21 ± 0.01	19.70 ± 0.06	1.03 ± 0.29	2.53	40.74
J25	24.11 ± 1.13	25	5.33 ± 0.03	10.36 ± 0.06	0.67 ± 0.11	1.52	43.93
J26	31.56 ± 0.17	25	4.63 ± 0.01	27.29 ± 0.39	0.80 ± 0.05	1.79	44.71
J27	32.41 ± 0.40	25	4.60 ± 0.01	20.55 ± 0.12	1.03 ± 0.13	1.71	60.19
J28	32.56 ± 0.33	25	4.94 ± 0.08	11.35 ± 0	1.03 ± 0.17	1.47	70.18
J29	34.21 ± 0.31	25	5.14 ± 0.05	8.07 ± 0.01	1.19 ± 0.08	1.76	67.43
J30	29.19 ± 0.42	25	4.89 ± 0.03	15.77 ± 0.04	0.62 ± 0.21	1.29	47.90
							
Thailand							
T1	35.46 ± 0.02	25	4.89 ± 0.05	10.26 ± 0	1.21 ± 0.1	2.16	56.09
T2	37.90 ± 0.22	25	5.11 ± 0.02	8.93 ± 0.04	0.70 ± 0.07	1.63	43.02
T3	34.78 ± 0.06	25	4.89 ± 0.08	9.34 ± 0.05	0.81 ± 0.07	1.79	45.36
T4	34.29 ± 0.07	25	4.92 ± 0	10.19 ± 0.03	0.60 ± 0.02	1.61	37.16
T5	36.41 ± 0.18	25	5.12 ± 0.11	6.43 ± 0.02	0.59 ± 0	1.32	44.57
T6	33.66 ± 0.68	25	5.10 ± 0	8.74 ± 0.07	0.60 ± 0	1.65	36.30
T7	35.44 ± 0.06	25	5.22 ± 0.03	6.09 ± 0.01	0.63 ± 0.19	1.43	43.91
T8	35.81 ± 1.14	25	5.13 ± 0.03	9.25 ± 0.01	0.95 ± 0.02	2.24	42.34
T9	32.91 ± 0.09	25	5.25 ± 0.06	4.42 ± 0.05	0.76 ± 0.03	1.44	52.91
T10	34.92 ± 0.34	25	5.17 ± 0.03	5.41 ± 0.02	0.52 ± 0.06	1.21	43.01
T11	27.51 ± 0.22	25	5.15 ± 0.01	9.11 ± 0.01	0.43 ± 0.01	1.2	35.91
							
Vietnam							
V1	30.20 ± 0.05	25	4.91 ± 0.09	16.49 ± 0.06	2.01 ± 0.06	2.94	68.26
V2	35.00 ± 4.01	25	5.10 ± 0.09	14.76 ± 0.05	1.31 ± 0.15	2.95	44.38
China, C1	27.46 ± 0.20	23	5.17 ± 0.04	6 ± 06 ± 0.03	0.69 ± 0.27	1.36	50.70
Philippine, P1	28.13 ± 0.27	25	5.13 ± 0.02	1.87 ± 0.02	0.30 ± 0.05	0.49	61.05
Italy, I1	30.05 ± 0.09	25	4.93 ± 0	11.73 ± 0.07	0.70 ± 0.62	1.45	48.22

Data are presented as the mean ± SD.

All measurements were done in triplicate, except for salinity and total nitrogen.

## 2 Experimental design, materials and methods

### 2.1 Design

Data are presented for the following numbers of different commercial fish sauce products: 30 sauces produced in Japan; 11 sauces from Thailand; two sauces from Vietnam; and one from each of the Philippines, China, and Italy (Table 1). For each fish sauce product, analysis was performed in triplicate.

### 2.2 Materials

Data for 46 fish sauces are presented. The ingredients of each product described on the product label are summarized in Table 1. For the analysis, the products were assigned product IDs, as follows: J1–J30 for the 30 Japanese products; T1–T11 for the Thai products; V1 and V2 for the Vietnamese products; and P1, C1, and I1 for the Filipino, Chinese, and Italian products, respectively. All fish sauce products were purchased in a local market in Tokyo, Fukuoka, or Abashiri (Japan).

### 2.3 Solid contents assay

To determine the dried solid content of the fish sauce products, ca. 2 g of fish sauce sample was applied to a moisture analyzer (MX-50; A&D, Japan). The measurements were conducted at 130 °C for 20 min, as described in Ref. [6].

### 2.4 Salinity and pH measurements

The salinity and pH of the fish sauce products were determined using a salt meter (B-721; HORIBA, Japan) and a pH meter (D-52; HORIBA), respectively.

### 2.5 Total acidity assay

Total acidity was determined by a titration assay. Briefly, 10 g of fish sauce samples were diluted up to 100 mL with distilled water. Acid content in 10 mL of the diluted sample was determined by titration with 0.1 M NaOH, with 1% (w/v) phenolphthalein solution as a pH indicator.

### 2.6 Amino acid content determination

The total nitrogen content was determined using SUMIGRAPH NC-220F analyzer (Sumika Chemical Analysis Service, Japan) [7].

Amino nitrogen content was determined using the formol titration method [8]. Briefly, 5 mL of the fish sauce sample was diluted up to 250 mL with distilled water. For the first titration, all of the diluted sample was titrated to pH 8.5 with 0.01 M NaOH. For the second titration, 20 mL of formaldehyde solution (pH 8.5) was added to the diluted sample, and then titrated to pH 8.5 with 0.1 M NaOH. The volume of base consumed in the first and second titration was used for calculating the amino nitrogen content [8].

The amino nitrogen to total nitrogen ratio, i.e., a value of amino nitrogen divided by total nitrogen, was used as an index of protein-to-amino acid conversion rate.
