# Supplementary material for: Data on the chemical properties of commercial fish sauce products
Source: Data Brief. 2017 Oct 14;15:658–64. doi: 10.1016/j.dib.2017.10.022 (PMC5671479; doi:10.1016/j.dib.2017.10.022)
Supplement: Supplementary file 1 — Transparency document [file mmc1.docx]

**Conflicts of interest:** none.
